# The *rulB* gene of plasmid pWW0 is a hotspot for the site-specific insertion of integron-like elements found in the chromosomes of environmental *Pseudomonas fluorescens* group bacteria

**DOI:** 10.1111/1462-2920.12345

**Published:** 2014-01-07

**Authors:** Glenn Rhodes, Hester Bosma, David Studholme, Dawn L Arnold, Robert W Jackson, Roger W Pickup

**Affiliations:** 1 Centre for Ecology and Hydrology, Lancaster Environment CentreLibrary Avenue, Bailrigg, Lancaster, LA1 4AP, UK; 2 Rijksuniversiteit GroningenPO Box 72, 9700 AB, Groningen, The Netherlands; 3 Geoffrey Pope Building, College of Life and Environmental Sciences, University of ExeterStocker Road, Exeter, EX4 4QD, UK; 4 Centre for Research in Biosciences, University of the West of EnglandBristol, BS16 1QY, UK; 5 School of Biological Sciences, University of ReadingReading, RG6 6UR, UK; 6 Division of Biomedical and Life Sciences, Faculty of Health and Medicine, Lancaster UniversityLancaster, LA1 4YQ, UK

## Abstract

The *rulAB* operon of *Pseudomonas* spp. confers fitness traits on the host and has been suggested to be a hotspot for insertion of mobile elements that carry avirulence genes. Here, for the first time, we show that *rulB* on plasmid pWW0 is a hotspot for the active site-specific integration of related integron-like elements (ILEs) found in six environmental pseudomonads (strains FH1–FH6). Integration into *rulB* on pWW0 occurred at position 6488 generating a 3 bp direct repeat. ILEs from FH1 and FH5 were 9403 bp in length and contained eight open reading frames (ORFs), while the ILE from FH4 was 16 233 bp in length and contained 16 ORFs. In all three ILEs, the first 5.1 kb (containing ORFs 1–4) were structurally conserved and contained three predicted site-specific recombinases/integrases and a *tetR* homologue. Downstream of these resided ORFs of the ‘variable side’ with structural and sequence similarity to those encoding survival traits on the fitness enhancing plasmid pGRT1 (ILE_FH__1_ and ILE_FH__5_) and the NR-II virulence region of genomic island PAGI-5 (ILE_FH__4_). Collectively, these ILEs share features with the previously described type III protein secretion system effector ILEs and are considered important to host survival and transfer of fitness enhancing and (a)virulence genes between bacteria.

## Introduction

Bacteria within the genus *Pseudomonas* are found in a wide range of terrestrial and aquatic natural and clinical environments and demonstrate remarkable metabolic and physiological versatility including the potential for pathogenicity (Morris *et al*., 2000; 2007; 2008; Riffaud and Morris, 2002). This has been particularly illustrated by sequenced genomes (Rodríguez-Palenzuela *et al*., 2010; Ortet *et al*., 2011; Ramírez-Díaz *et al*., 2011; Winsor *et al*., 2011; Yu *et al*., 2011; Patel *et al*., 2012). These have revealed the extent of the horizontal transfer of mobile genetic elements such as phage, transposons and insertion sequences, and genomic and pathogenicity islands (Roy *et al*., 2010; Martinez *et al*., 2012; Morales-Espinosa *et al*., 2012; Tang *et al*., 2012; Wu *et al*., 2012), and the mosaic nature of bacterial genomes in general (Hall, 2012; Marttinen *et al*., 2012).

The *rulAB* operon in *Pseudomonas* spp. has been shown to confer fitness traits including ultraviolet (UV) tolerance on its host (Sundin *et al*., 1996; Gibbon *et al*., 1999) and to be involved in the SOS response and the growth advantage in stationary phase phenotype (Tark *et al*., 2005; Kivisaar, 2010). The operon is common to both the chromosomes and plasmids of pseudomonads (Sundin *et al*., 2000; Zhao *et al*., 2005; Cazorla *et al*., 2008). In the latter, it is usually located close to transfer or mating pair formation encoding regions in the core backbone, ensuring that it is one of the first regions transferred during conjugation (Gibbon *et al*., 1999).

Analysis of *Pseudomonas* genomes demonstrated that *rulAB* is common in an intact or an interrupted form. Its function and benefit to bacterial hosts is still relatively poorly understood (Jackson *et al*., 2011). Arnold and colleagues (2001) found that the avirulence gene *avrPpiA1* resided in a 4.3 kb region that interrupted the *rulB* gene in *P. syringae* pv*. pisi* and concluded that the *rulB* gene may be a hotspot for insertion of mobile regions of DNA. Interruption of the *rulB* gene by integration of integron-like elements (ILEs) led to the postulation that the *rulAB* promoter controls the expression of integrase under the regulation of LexA repressor protein (a LexA binding site can be found upstream of *rulAB*) (Jackson *et al*., 2011). This association is broad, with similar disruptions of *rulAB*-related DNA repair genes *rumAB*, *umuDC*, *impAB*, *mucAB*, *samAB* and *ruvAB* in a range of bacteria including the insertion of the SXT conjugative element that confers pathogenicity and is embedded in *rumB* of *Vibrio cholera* (Hochhut *et al*., 2001).

The 117 kb plasmid pWW0 is the archetypal plasmid of the IncP-9 group, a family of large self-transmissible plasmids found mainly in pseudomonads that harbour genes for antibiotic and heavy metal resistance and the biodegradation of mono-aromatic and polyaromatic compounds (toluene/xylenes and naphthalene) (see Sevastsyanovich *et al*., 2008). In pWW0, these genes are harboured within the 70 kb transposon Tn*4653*, with the remainder of the plasmid containing the core backbone functions. Although classed as a narrow host range plasmid, pWW0 can transfer at frequencies as high as 10^−1^ to 1 transconjugant per recipient cell between pseudomonads (Nakazawa, 1978; Ramos *et al*., 1987) and can transfer to enterobacteriaceae at lower frequencies (see Ramos *et al*., 1997). It also has the capability for retrotransfer (Ronchel *et al*., 2000). Carriage of pWW0 has been shown to be beneficial to host bacteria not only through traits encoded by the accessory genes within Tn*4653* but also from those encoded by the *rulAB*-homologue genes (termed *ruvAB*; Greated *et al*., 2002) within the core backbone. In pWW0, these genes are located between positions 5405–7034 and have been shown to encode a DNA polymerase Pol V homologue that significantly increases the evolutionary fitness of the *P. putida* host bacteria during prolonged nutritional starvation (Tark *et al*., 2005).

In the present study, we report for the first time the active integration of a group of related ILEs from environmental *Pseudomonas* spp. isolates into plasmid pWW0 and show that insertion into *rulAB* operon and its homologues in other genera is potentially of key importance to the adaptation and survival of these bacteria.

## Results

### Discovery of a novel ILE

During an investigation of plasmid-encoded copper resistance in environmental pseudomonads recovered in a previous study (Pickup, 1989), we attempted to cure native plasmids from these strains by incompatibility using the IncP-9 toluene-degrading plasmid pWW0. After conjugation between *P. putida* PaW340 (pWW0) and environmental isolate FH1 (Table 1), and subsequent verification of FH1 (pWW0) transconjugants by restriction digest analysis of pWW0_FH1_, we observed that plasmid pWW0 had acquired an extra region of DNA and that this process was repeatable. Restriction mapping showed the insert to be around 10 kb in size; the region was subsequently cloned on a *Pst*I fragment into vector pBR325, and the recombinant plasmid designated pFBA1001 (not shown). This region was subsequently shown by DNA hybridization against genomic DNA from plasmid-cured FH1 to be chromosomally located (not shown).

**Table 1:** Bacterial strains and plasmids

Strain	Relevant characteristics	Source/reference
Environmental pseudomonads		
FH1 (isolated in 1985)	Chromosomally located ILE_FH1_; Km^S^, Sm^S^	This study
FH2 (isolated in 1995)	Chromosomally located ILE_FH2_; Km^S^ Sm^S^	This study
FH3 (isolated in 1995)	Chromosomally located ILE_FH3_; Km^S^ Sm^S^	This study
FH4 (isolated in 1995)	Chromosomally located ILE_FH4_; Km^S^ Sm^S^	This study
FH5 (isolated in 1995)	Chromosomally located ILE_FH5_; Km^S^ Sm^S^	This study
FH6 (isolated in 1995)	Chromosomally located ILE_FH6_; Km^S^ Sm^S^	This study
Control strains/constructs		
*Pseudomonas putida* PaW340	Sm^R^; trp−	DSM 2112
*P. putida* PaW340 (pWW0)	Sm^R^; TOL; trp−	Franklin and Williams (1980)
*P. putida* EEZ15 (pWW0::Km^R^)	Sm^S^; Km^R^	Ramos-Gonzalez and colleagues (1994)
*P. putida* PaW340 (pWW0::Km^R^)	Sm^R^; TOL, Km^R^; trp−	This study
*P. putida* PaW85 (pWW0ΔrulAB::Km^R^)	Sm^S^; TOL; Km^R^	Tark and colleagues (2005)
*P. putida* PaW340 (pWW0ΔrulAB::Km^R^)	Sm^R^; TOL; Km^R^; trp−	This study
*Escherichia coli* HB101 (pFBA1001)	PstI fragment containing ILE_FH1_ and truncated rulAB ends cloned into pBR325; Sm^R^, Tc^R^, pro-, leu-, thy-.	This study
*P. putida* PaW340 (pWW0::Km^R^::ILE_FH1_)	pWW0 located ILE_FH1_ Sm^R^; TOL, Km^R^; trp−	This study
*P. putida* PaW340 (pWW0::Km^R^::ILE_FH4_)	pWW0 located ILE_FH4_ Sm^R^; TOL, Km^R^; trp−	This study
FH1 (pWW0::Km^R^::ILE_FH1_)	pWW0 located ILE_FH1_ Sm^R^; TOL, Km^R^; trp−	This study
FH2 (pWW0::Km^R^::ILE_FH2_)	pWW0 located ILE_FH2_ Sm^R^; TOL, Km^R^; trp−	This study
FH3 (pWW0::Km^R^::ILE_FH3_)	pWW0 located ILE_FH3_ Sm^R^; TOL, Km^R^; trp−	This study
FH4 (pWW0::Km^R^::ILE_FH4_)	pWW0 located ILE_FH4_ Sm^R^; TOL, Km^R^; trp−	This study
FH5 (pWW0::Km^R^::ILE_FH5_)	pWW0 located ILE_FH5_ Sm^R^; TOL, Km^R^; trp−	This study
FH6 (pWW0::Km^R^::ILE_FH6_)	pWW0 located ILE_FH6_ Sm^R^; TOL, Km^R^; trp−	This study

Km, kanamycin; ^R^, resistant; ^S^, sensitive; Sm, streptomycin.

The 10 kb region of pWW0_FH1_ in pFBA1001 was sequenced; and a complete assembly was constructed. Putative open reading frames (ORFs) were identified, and the DNA and protein sequences within this region were aligned with sequences in the databases. The *Pst*I fragment was 10 165 bp in length and was flanked on either side by 480 and 282 bp of a disrupted *rulB* gene. The *rulB*-flanked region was therefore 9403 bp in length and contained eight ORFs (Table 2). Alignments revealed that all eight ORFs had the closest nucleotide and protein identity with ORFs 26–35 in plasmid pGRT1 of *P. putida* DOT-T1E that is tolerant to high concentrations of toluene via efflux pumping (Molina *et al*., 2011) (Table 2). Notably, ORFs 1–3 were phage integrases/site-specific recombinases. The predicted protein of ORF1 possessed the C-terminal R-H-R-Y motif of tyrosine recombinases and multidomains of XerC and XerD recombinases, and was therefore designated *xerD* (Supporting information Fig. S1). ORF2 and ORF3 were also putative site-specific recombinases that possessed the INT_REC_C conserved domain (not shown).

**Table 2:** Predicted ORFs on FH1 integron-like element in relation to plasmid pGRT1 in *P. putida* DOT-T1E

ORF	Name	Protein length (aa)	Direction	Amino acid (aa) identity to ORFs on pGRT1^a^	Predicted protein function
1	*xerD*	385	←	ORF26; 99% in 385 aa	XerD-like phage integrase
2	int/rec	525	←	ORF27; 99% in 525 aa	Hypothetical protein with INT_REC_C conserved domain
3	int/rec	535	←	ORF30; 99% in 452 aa	Site-specific recombinase/phage integrase family protein with INT_REC_C conserved domain
4	*tetR*	138	←	ORF31; 99% in 138 aa	TetR family transcriptional regulator-like protein
5	*sdiA*	320	→	ORF32; 96% in 320 aa	SdiA-regulated motif containing protein on plasmid pGRT1 shown to be a modulator of the TtgGHI efflux pump in host *P. putida* DOT-T1E
6	*dksA*	117	→	ORF33; 98% in 117 aa	Hypothetical protein, DnaK suppressor-like (signal transduction mechanisms)
7	*uspA*	283	→	ORF34; 96% in 283 aa	UspA protein (universal stress response protein) on plasmid pGRT1 shown to be involved in UV response and after mild induction to increase tolerance to toluene in *P. putida* DOT-T1E
8	*sulP*	495	→	ORF35; 99% in 495 aa	Sulphate permease with STAS domain (sulphate transporter and anti-sigma factor) to be involved in siderophore production in *P. putida* DOT-T1E

a Accession number HM626202.

The only significant difference between the pFBA1001 element and its counterpart region on pGRT1 was the presence in pGRT1 of an IS*4*-like transposase (ORF29) that is absent from pFBA1001. In pGRT1, this transposase divides ORF28 and ORF30 (also both predicted to encode site-specific recombinases), and its *in silico* deletion from pGRT1 results in the same sequence found in ORF3 (*int/rec*) on pFBA1001, suggesting the possibility of an insertion event (not shown). As in pFBA1001, ORFs 26–35 in pGRT1 are flanked by *ruvAB* (*rulAB*) genes (ORFs 25 and 36) homologous with *rulAB* of pWW0. In addition, the region is oriented in the same way as in pFBA1001.

The sequence of the ORF5 predicted protein shares 96% identity with that encoded by ORF32 on pGRT1 and was predicted to be an SdiA-regulated motif protein involved in modulation of the TtgGHI efflux pump (Molina *et al*., 2011). Similarly, ORF7 that shares 96% protein sequence identity with pGRT1 ORF34 was predicted to encode a universal stress response protein UspA, which in the latter conferred a two-order of magnitude survival advantage to toluene shock after moderate exposure to toluene stress (Molina *et al*., 2011). ORF8 was homologous to ORF35 on pGRT1 and was predicted to encode a sulphate permease that has been shown to be involved in siderophore production (possibly via the release of a pseudobactin-like siderophore (see Molina *et al*., 2011). Collectively, the presence of a *xerD* integrase, *tetR* gene and other possible fitness-enhancing traits in the mobile region from FH1 were suggestive of an integron-like structure. For this reason, the FH1 element was designated an ILE.

### The FH1 ILE is diverse and associated with UV-resistance gene *rulB*

**Fig. 1:**
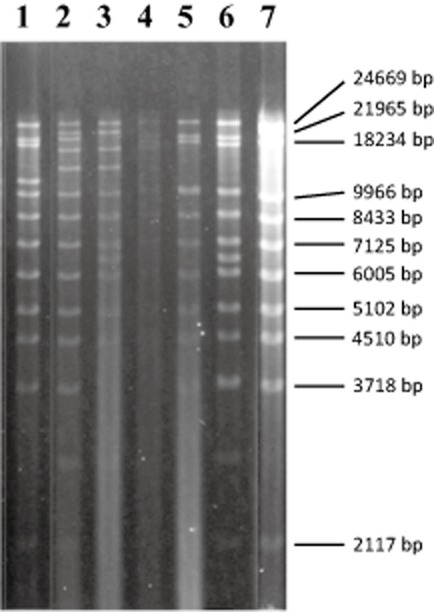
RFLP profiles of *Hin*dIII digested pWW0 plasmid variants from strains FH1–FH6. Lanes 1–6 = pWW0_FH__1–__FH__6_. Lane 7 = pWW0. The size of fragments generated from *in silico* digestion of pWW0 are shown for comparison.

The distribution of ILEs in the environment was assessed in naturally occurring pseudomonads recovered from Copper Mines Valley in the English Lake District (Cumbria, UK). From hundreds of colony-forming units (CFU) initially isolated on *Pseudomonas* selective agar, 800 presumptive pseudomonad isolates were purified. Isolates were not characterized further and, because of the isolation media used, are not guaranteed to be independent isolates. Purified isolates were screened for similar ILEs by colony hybridization using the entire pFBA1001 10 kb *Pst*I restriction fragment as a DNA probe. This resulted in 11 positive signals (1.4%; not shown). Conjugation of hybridization positive strains with *P. putida* PaW340 (pWW0) resulted in the insertion of regions of approximately 9–16 kb in size into pWW0 in 5 of the 11 isolates. In each case, the frequency of plasmid transfer ranged between 10^−4^ and 10^−2^ per recipient. Restriction fragment length polymorphism (RFLP) profiling showed that all altered pWW0 plasmids were different, and it was therefore assumed that all six ILEs were different (Fig. 1). The original bacterial isolates containing these ILEs were designated strains FH1–FH6 (Table 1), and the altered pWW0 plasmids that arose after mating with *P. putida* PaW340 (pWW0) were named pWW0::ILE_FH1–6_.

**Fig. 2:**
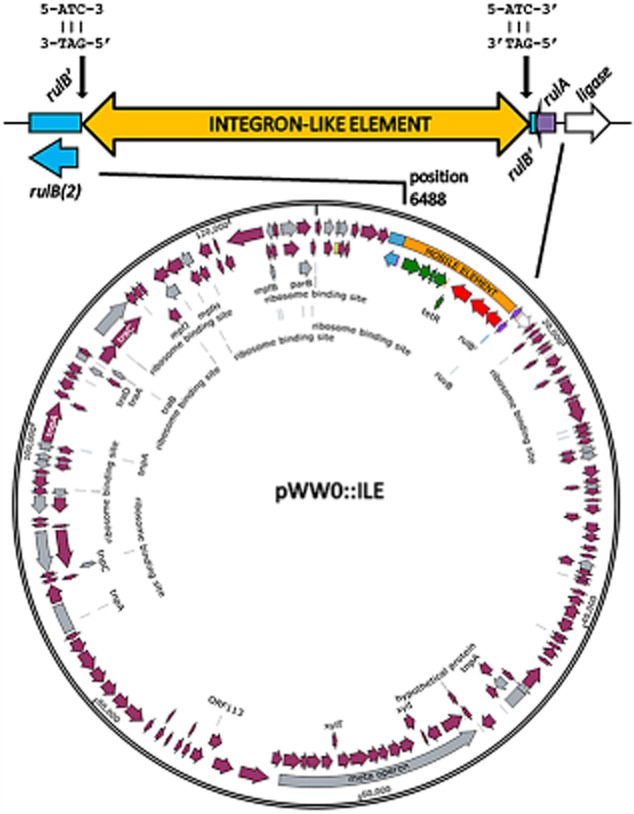
Insertion point and orientation of ILEs in pWW0. ILEs (orange) inserted into the *rulB* gene (blue) at position 6488 on pWW0, generating the truncated *rulB′* and a new predicted ORF *rulB(2)*. The direct repeat created by insertion is illustrated.

Restriction mapping of plasmids pWW0_FH2–6_ using the published sequence of pWW0 as a reference (Greated *et al*., 2002) suggested that as for pWW0_FH1_, insertion of the ILE in each case was also most likely into the *rulAB* operon. Based upon the position of ILE_FH1_ (from pWW0::ILE_FH1_), insertion into pWW0 at this point would result in an unaltered *rulA* gene, but with an interruption 123 bp into the *rulB* gene (herein referred to as *rulB'*). However, interruption at this point created an alternative ORF [*rulB*(*2*)] encoding a predicted protein of 345 aa with a start codon at original position 6440 (Fig. 2). Fine mapping and sequencing of the region in pWW0::ILE_FH1_ revealed the insertion of ILE_FH1_ into pWW0 occurred between positions 6488–6490 in the *ruvB* (*rulB*) gene generating a target repeat of 5′-GAT-3′ at the insertion site (Fig. 2).

### Specificity of ILE insertion into pWW0

The specificity of the integration was investigated by assessing insertion sites in plasmids pWW0::ILE_FH1–6_ by polymerase chain reaction (PCR) amplification using the primers described in Table 3. DNA from plasmids pWW0::ILE_FH1–6_ and the genomes of original strains FH1–FH6 and *P. putida* PaW340 (pWW0) was extracted, and amplification was performed (Table 4). Amplification of the intact *rulAB* region was successful from pWW0 DNA but not from FH1–6 genomic DNA or plasmids pWW0::ILE_FH1–6_. This confirmed that an intact pWW0-like *rulAB* was not carried in the genomes or in pWW0 transconjugants. Amplification of the region spanning the rulAB-xerD (590 bp) was positive for plasmids pWW0::ILE_FH1–6_ but negative for the genomes of original isolates and *P. putida* PaW340. This indicated that in each case, the *rulAB* operon had been interrupted by insertion and that a region found in ORF1 (*xerD*) on the integrating region was common to all transconjugants. This was confirmed with the amplification of a region of the *xerD* gene from plasmids pWW0::ILE_FH1–6_. These findings also showed that the six ILEs had interrupted *rulAB* in the same orientation (see Fig. 2). However, at the right-hand end of the ILEs, there was variability as primers that spanned the intergenic *rulB*-*sulP* junction amplified from plasmids pWW0::ILE_FH1_ and pWW0::ILE_FH5_ only.

**Table 3:** PCR primers and assay details

Primer name	Amplifies	Sequence 5′-3′	Expected product size
rulABFP	intact *rulAB region*	TGGCGTATGTCGATAACCAG	423 bp
rulABRP		CAATTCCCCGTACAAGGTGT	
xerDFP	*xerD* region	AGCAGCGCAACCTGATAACT	501 bp
xerDRP		GCCTGCCTTCATTAGTCAGC	
rulAB-xerDFP	*rulAB-xerD* flank	TGGCGTATGTCGATAACCAG	590 bp
rulAB-xerDRP		GTACAGACGCCGTCCATAGG	
rulB-sulPFP	*rulB′-sulP* flank	TTATTTTGCTGTGCGCTTTG	513 bp
rulB-sulPRP		CAATTCCCCGTACAAGGTGT	

**Table 4:** Assessment of the specificity of ILE integration by PCR amplification of ILE-specific regions in original host genomes and on pWW0 in transconjugants

	Amplification product (primer set)			
Strain/DNA	rulAB	xerD	rulAB-xerD	rulB′-sulP
pWW0	+	−	−	−
FH1	−	+	−	−
pWW0::ILE_FH1_	−	+	+	+
FH2	−	+	−	−
pWW0::ILE_FH2_	−	+	+	−
FH3	−	+	−	−
pWW0::ILE_FH3_	−	+	+	−
FH4	−	+	−	−
pWW0::ILE_FH4_	−	+	+	−
FH5	−	+	−	−
pWW0::ILE_FH5_	−	+	+	+
FH6	−	+	−	−
pWW0::ILE_FH6_	−	+	+	−

PCR products obtained from the rulAB-xerD and rulB-sulP primer pair amplifications were sequenced resulting in sequences for each end of the region inserted into pWW0::ILE_FH1_ and pWW0::ILE_FH5_. In each case, it was demonstrated that insertion occurred at exactly the same position on pWW0 and generated a 5′-GAT-3′ direct repeat at the insertion point (Fig. 2).

The importance of this insertion site to the movement and integration of ILEs was tested by conjugation between strains FH1, FH4 and FH5, and *P. putida* PaW340 (pWW0Δ*rulAB*::Km^R^) by filter matings. From each of these matings, 20 transconjugants were screened for insertion into pWW0 by carrying out the xerD PCR on extracted plasmids (because the more specific rulAB-xerD PCR assay could not be used due to loss of the forward primer locus). Amplification did not occur (positive control DNA amplified as expected) suggesting that integration did not take place either at this original site or elsewhere on pWW0 (not shown). In matings between FH1, FH4 and FH5, and *P. putida* PaW340 with the intact *rulAB* carrying plasmid (pWW0::Km^R^), this frequency of integration of ILEs was between 20% and 85% (not shown).

### The sequence and location of the ILEs in the genomes of FH1, FH4 and FH5

**Fig. 3:**
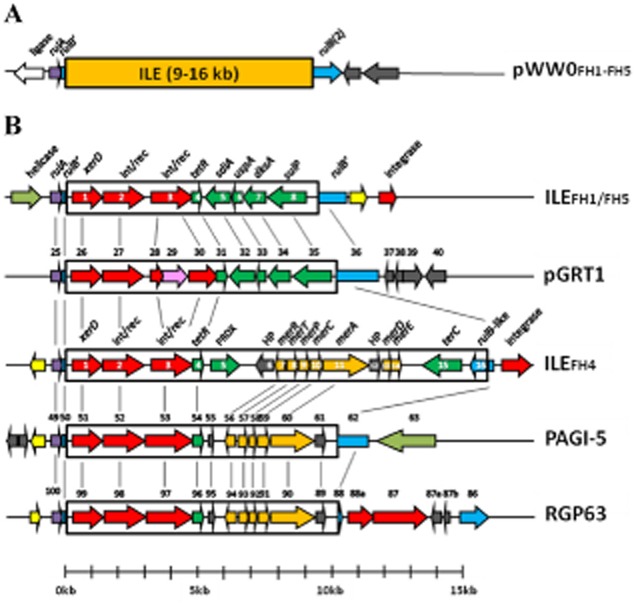
The structure of ILEs from FH1/FH5 and FH4 aligned with regions of closest similarity. (A) The general structure of ILEs inserted into *rulB* on pWW0 with ORFs flanking the insertion point on pWW0 is illustrated. (B) The detailed structure of chromosomally located ILE_FH__1–__FH__5_ alongside predicted ILEs in *P. putida* DOT-1E plasmid pGRT1 and *P. aeruginosa* genomic islands PAGI-5 and RGP63. ILEs are shown inside black rectangles with interrupted flanking *rulB*-like regions (light blue). Related regions are linked by adjoining black lines. When not specifically indicated, other colours indicate the following: Blue: *rulB*-like regions; purple: *rulA*-like gene; red: site-specific recombinase/phage integrase; pink: transposase, dark green, fitness-related; pale green: helicase; orange: mercury resistance genes; grey: hypothetical proteins (HP); yellow: hypothetical proteins with domains of unknown function. Predicted ORFs for ILE_FH__1/5_ and ILE_FH__4_ are numbered inside arrows, whereas those of relatives are shown above the sequence and are numbered in accordance with deposited sequences. ORFs 87a and b and ORF 88a in RGP63 are predicted in the present study and not in the original genome sequence. The sequences of pGRT1, PA7 (RGP63) and PAGI-5 are HM626202, CP000744 and EF611301 respectively.

The sequence of the ILE on pWW0::ILE_FH1_ ascertained from pFBA1001 elucidated the structure and location on pWW0 but did not confirm its location or structure in the genome of strain FH1. To better understand this, we obtained the draft genome sequences of strains FH1, FH4 and FH5, which based on RFLP profile, data represented three different ILEs. The ILEs within strains FH1, FH4 and FH5 were located in the draft sequences by alignment using the ILE sequences inserted into *rulB* on pWW0 in each strain. Interestingly, in the case of all three strains, ILEs were located inside a chromosomal *rulB* gene within a disrupted *rulAB*-like operon that differed to *rulAB* on pWW0 (see Fig. 3).

It was as shown that the DNA sequence of ILE_FH5_ shared 97% nucleotide identity with that of ILE_FH1_, was also 9403 bp in length and contained ORFs 1–8 that shared at least 93% protein sequence identity with those of ILE_FH1_ (Fig. 3).

In contrast, the ILE_FH4_ differed in that it was 16 233 bp in length and carried 16 predicted ORFs (Fig. 3 and Table 5). The first four ORFs encoded predicted proteins identical with those from ORFs 1–4 in ILE_FH1_ and ILE_FH5_ (*xerD* to *tetR*). This was reflected in the fact there was 99% identity at the nucleotide level over the first 5.1 kb between ILE_FH1_ and ILE_FH5_, and 84% with that of ILE_FH4_. However, immediately downstream of the *tetR* gene, the sequences diverged, and in ILE_FH4_, the remaining 11.1 kb contained 12 predicted ORFs unrelated to those in the right-hand side of ILE_FH1_ and ILE_FH5_. This region contained ORFs homologous to those of the ubiquitous mercury-resistance *mer* operons (*merR*, *merT*, *merP*, *merC*, *merA*, *merD* and *merE*) with closest nucleotide identity (92% across the 4.3 kb in which these genes were located) to the same genes in Tn*5041* (not shown) (Kholodii *et al*., 2002). Downstream of the *mer* genes was ORF15, predicted to encode an integral membrane protein (TerC family), associated with tellurium resistance. Interestingly, ORF16 was predicted to encode another RulB-like protein; however, it was orientated in the opposite direction to the chromosomal *rulAB* operon interrupted by ILE_FH4_ itself (Fig. 3).

**Table 5:** Predicted ORFs on the FH4 integron-like element

ORF	Name	Protein length (aa)	Direction	Amino acid (aa) identity to informative database match (accession number)
1	*xerD*	385	→	99% in 385 aa; ORF26 in plasmid pGRT1, XerD-like phage integrase (HM626202).
2	int/rec	525	→	99% in 525 aa; ORF27 in plasmid pGRT1, hypothetical protein with INT_REC_C conserved domain (HM626202).
3	int/rec	535	→	99% in 452 aa; ORF30 in plasmid pGRT1, site-specific recombinase/phage integrase family protein with INT_REC_C conserved domain (HM626202).
4	*tetR*	138	→	99% in 138 aa; ORF31 in plasmid pGRT1, TetR family transcriptional regulator-like protein (HM626202).
5	PRDX	360	→	89% in 360 aa; peroxiredoxin in *Pseudomonas* sp. GM49 (ZP_10658778).
6	HP	229	←	90% in 41 aa; hypothetical protein with sequence similarity to a region of Tn*5041* in *Pseudomonas* sp. (CAC80074).
7	*merR*	139	←	97% in 139 aa; putative transcriptional regulator MerR in *P. aeruginosa* (NCGM1179).
8	*merT*	134	→	78% in 104 aa; mercuric transport protein MerT in *P. aeruginosa* PA7 (ABR82023)
9	*merP*	134	→	99% in 91 aa; putative MerP protein component of transporter in *P. mandelii* JR-1 (ZP_11114267)
19	*merC*	144	→	90% in 143 aa; putative MerC superfamily protein in *P. mandelii* JR-1 (ZP_11114268) and *P. aeruginosa* ATCC 700888 (ZP_15625973)
11	*merA*	581	→	95% in 560 aa: mercuric reductase protein MerA in *P. mandelii* JR-1 (ZP_11114269)
12	HP	139	→	83% in 138 aa; Hypothetical protein in *Pseudomonas* sp. (CAC80080)
13	*merD*	120	→	100% in 120 aa: mercuric resistance transcriptional repressor MerD, MerR family in *P. mandelii* JR-1 (ZP_11114271)
14	*merE*	79	→	96% in 77 aa; MerE superfamily mercury resistance protein in *P. mandelii* JR-1 (ZP_11114272)
15	*terC*	515	←	96% in 515 aa; TerC superfamily integral membrane protein in *Pseudomonas* sp. UW4 (YP_007029200)
16	*rulB*-like	160	←	60% in 104 aa; putative ImpB/MucB/SamB/RulB family protein of DUF4113 superfamily in *P. stutzeri* TS44 (ZP_1447253)

### Effect of insertion of ILEs into pWW0*_rulB_* on UV tolerance

The effect of ILE insertion into pWW0*_rulB_* on host strain tolerance to UV was assessed in *P. putida* PaW340 hosts. In three independent experiments, the growth of strains *P. putida* PaW340 (pWW0::Km^R^), and *P. putida* PaW340 (pWW0::Km^R^::ILE_FH1_) and *P. putida* PaW340 (pWW0::Km^R^::ILE_FH4_) showed a 3 log reduction in growth after 30 s of exposure to UV (302 nm) compared with controls not exposed to UV (Supporting information Fig. S2). Plasmid-free PaW340 and PaW340 (pWW0Δ*rulAB*::Km^R^) both suffered 5 log reductions in CFU numbers after the same UV exposure (Supporting information Fig. S2). This suggested that insertion into *rulB* on pWW0 had no adverse effect on UV tolerance.

### ILEs associated with *rulB*-like genes are present in plant and animal pathogens, and encode known virulence and fitness factors

As ILE_FH1_ was shown to contain similar ORFs associated with fitness-conferring traits on pGRT1, we determined whether these ILEs have a wider significance by screening the genomes of other bacteria deposited in databases for their presence. Noteworthy was the homology and structural similarities that ILE_FH4_ shared with regions in the 75 kb *P. aeruginosa* PA7 genomic island RGP63 (Roy *et al*., 2010) and the 99 kb *P. aeruginosa* genomic island PAGI-5 (Battle *et al*., 2008). In each of these cases, the general structure of a truncated *rulAB'* operon flanking *int/rec* genes and *tetR* followed by *mer* genes was observed (Fig. 3). A similar structure, but lacking the *tetR* gene, was observed in the 123 kb *P. aeruginosa* plasmid pUM505 (Ramírez-Díaz *et al*., 2011). In pUM505, the overall structure differed due to interruption of the *mer* operon by a *tnpA* gene (Ramírez-Díaz *et al*., 2011). In the genomic island RGP63, the ILE_FH4_-like structure was located in a region spanning 10 kb between ORF88 (designated *umuC*) and ORF99 (designated *ruvB*). This 10 kb region has been shown previously to share homology with a 9.8 kb region in genomic island PAGI-5 (Roy *et al*., 2010). Further analysis of this relationship in the present study has shown that the homology in this region between RGP63 and PAGI-5 is 99% over a 9.9 kb region and that in PAGI-5 the region is also bound by flanking *rulB*-like sequences. Significantly, on PAGI-5, this 9.9 kb is located in NR-II that has been shown to contribute to the highly virulent phenotype of host strain *P. aeruginosa* PSE9 (Battle *et al*., 2008).

**Fig. 4:**
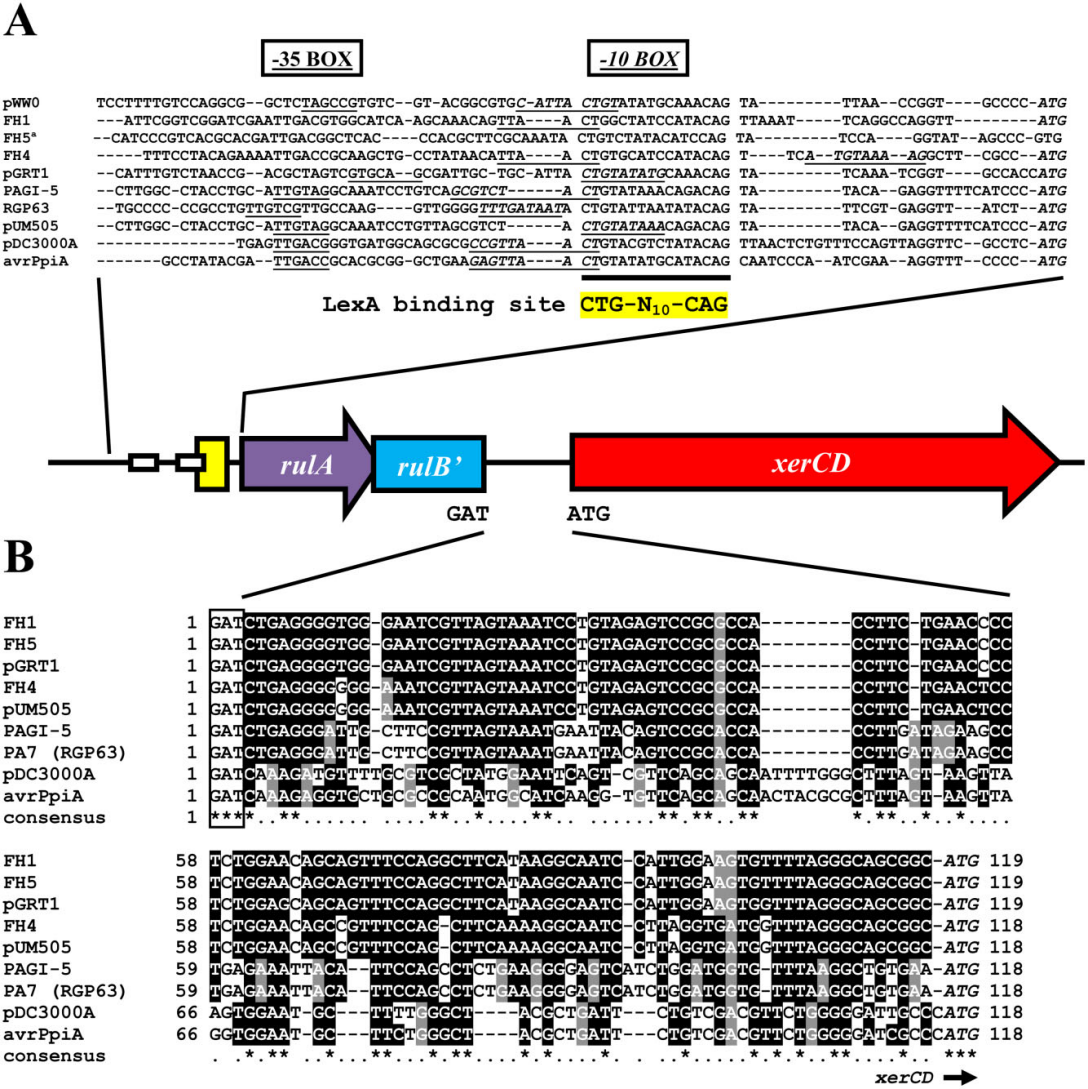
Alignment of intergenic regions found immediately upstream and downstream of *rulAB′* on integron-like elements.A. Alignment of the predicted promoter region and LexA binding site upstream of *rulA*. The conserved CTG-N_10_-CAG LexA binding site motif (yellow), the −35 box (bold and underlined) and the −10 box (underlined bold italics) are highlighted.B. The 118–119 bp intergenic region between the known 5′-GAT-3′ insertion point in pWW0 and the predicted ATG start codon of ORF1 (*xerC*/*xerD*) aligned with chromosomal locations in strains FH1, FH4 and FH5, and other close relatives. ^a^No ATG start codon for *rulA* in FH5 chromosome.

Comparison of the sequences of ILE_FH1_, ILE_FH4_ and ILE_FH5_ with proposed ILEs in pGRT1, PAGI-5, RGP63, pUM505 and another candidate region on the chromosome of *P. syringae* pv. *tomato* DC3000 showed that all share structural features with the recently proposed type III protein secretion system effector (T3SE) ILEs (Jackson *et al*., 2011). T3SE ILEs have T3SE gene(s) orientated so that the transcription is towards the 3′ end of the integrase gene and therefore not under the influence of the integrase P_c_ promoter. Although we have not identified T3SE genes on the ILEs here, this feature is shared with the integrated genes downstream of *tetR* in the ILE_FH1_ and ILE_FH5_, and on pGRT1, but not with all sequences downstream of *tetR* in FH4, pUMU505, PAGI-5 and RGP63 (Fig. 3). In addition, we have been unable to demonstrate the presence of a P_c_ promoter in the upstream integrase gene. However, even if present, its influence would not be exerted on *rulA* or disrupted *rulB'* that flank the element since they are transcribed in the opposite direction. In T3SE ILEs, insertion into the *rulAB* operon is considered likely to be under the influence of the LexA repressor because of a LexA binding region in the *rulAB* promoter (Jackson *et al*., 2011). Consistent with this, we found LexA1 binding sites with the characteristic CTG-N_10_-CAG motif upstream of *rulA* in each of the chromosomally located ILEs of FH1, FH4 and FH5 as well in plasmids pWW0, pGRT1 and genomic islands PAGI-5, RGP63 and pUM505 (Fig. 4A).

In T3SE-integrons, it was also observed that the integrase gene was situated less than 100 nucleotides downstream of the 5′ end of the truncated *rulB'* gene, and each case lacked its own upstream LexA or RpoD binding site (Jackson *et al*., 2011). In *P. syringae* pv. *tomato* DC3000 plasmid A and *P. syringae* pv. *pisi avrPpiA* chromosome site, both of which were described as carrying ‘complete’ T3SE ILEs, the integrase gene was 60 bp from the end of *rulB* (Jackson *et al*., 2011). More significantly, in the present study, we observed that the start codon of the *xerD* integrase gene was either 118 nt (ILE_FH4_) or 119 nt (ILE_FH1_ and ILE_FH5_) from the GAT point of insertion at the end of truncated *rulB* (*rulB'*; Fig. 4B). For the *avrPpiA1*-containing element and that on DC3000 plasmid A, both of which contained a predicted *rulB'* ORF, this GAT triad is also found 118 bp upstream of the integrase start codon ATG (Fig. 4B). This was also the case for the putative ILEs in PAGI-5, RGP63 and plasmid pUM505 (Fig. 4B). In pGRT1, the relationship with ILE_FH1/FH5_ was strengthened with the distance also being 119 nt (Fig. 4B). While we have no evidence regarding the specific site of integration in each of these other putative ILEs, we cannot rule out the potential importance of this observation to the integration of this family of ILEs in general.

### Analysis of the ILE insertion site in different genomes

To investigate the potential for insertion into *rulB*-like regions and the extent to which it may have already occurred in the genomes of other bacteria, we performed DNA alignments using 123 bp regions that spanned 60 bp on either side of the insertion site of both the intact and interrupted pWW0 *rulB* gene (Supporting information Fig. S3). The intact region of pWW0-*rulB* aligned with five sequences originating in catabolic plasmids (pND6-2, pDTG1, pNAH7, pNAH20 and KOPRI126573) from *Pseudomonas* spp. (Supporting information Fig. S3A). Five sequences of different origin to those earlier were identified with homology to the two 123 bp *rulB*-ILE junctions, of which four aligned with both ends. As previously, these four aligning sequences were from plasmids pGRT1, pUM505 and genomic islands PAGI-5 and RGP63. In each case, the pWW0 insertion point was preserved at the *xerD* side, and the 60 bp in the intergenic region between the insertion point and *xerD* contained three highly conserved regions including a 7 bp sequence (CTGAGGG) immediately inside the insertion point (Supporting information Fig. S3B). However, these conserved regions were not found in the proposed ILEs in pDC3000A or in that harbouring the *avrPpiA* gene (Fig. 4B). At the right-hand side of the element, the 60 bp of the intergenic region was similarly conserved despite ORFs on this side being variable (Supporting information Fig. S3C). In each of the aligning DNAs the 60 bp on the outside of each of these intergenic regions was shown to be a *rulB*-like sequence indicative of an insertion event having already taken place.

Despite the sequence conservation at each end of the mobile regions, repeat regions that might be involved in movement of the element were not found, and the significance of each of these conserved regions is not presently understood.

### Phylogenetic analysis of the ILEs and their host strains

Strains FH1–FH6 were identified as *Pseudomonas fluorescens* by API20E biochemical tests (not shown). Alignment of 797 bp of the *gyrB* gene obtained from the draft genomes of FH1, FH4 and FH5 with their closest relatives is shown in Supporting information Fig. S4. All three strains were placed within the *P. fluorescens* species complex, with FH1 and FH5 being located in the *P. fluorescens* subgroup with closest relatives being *P. extremorientalis* LMG 1965^T^ (FH1) and *P. libaniensis* CIP 105460^T^ (FH5). Strain FH4 was placed within the *P. gessardi* subgroup with *P. brenneri* DSM 15294^T^ as its closest relative (Mulet *et al*., 2010). This phylogenetic grouping corresponded well to the relationship of the ILEs characterized here, whereby ILE_FH1_ and ILE_FH5_ were very closely related but different to ILE_FH4_. Further analysis of the three phage integrase/site-specific recombinase genes and comparison with those of other ILEs confirmed this (Supporting information Fig. S5) and suggested that ILEs may have been associated with different clades of *P. fluorescens* group bacteria for some time.

## Discussion

A key objective in understanding bacterial evolution is to gain insight to the various mechanisms underpinning genotypic and phenotypic changes. By examining the outcome of plasmid conjugation events between environmental *Pseudomonas* bacteria, we have discovered a new set of genetic elements, reporting for the first time the observation of active site-specific integration of a novel and related group of ILEs into the *rulAB* operon on plasmid pWW0. The environmental pseudomonads described here were isolated between 18 and 28 years ago, and from a relatively small sample of cultured pseudomonads. The frequency of confirmed ILEs within this sample group (*n* = 800) was 0.75%, which suggests that the number of this family of ILEs alone in the environment is likely to be large and of significance to the transfer of fitness or virulence/avirulence traits between bacteria. Based on DNA and protein homology and similar structural features, we have proposed that other members of this group exist in genomes and plasmids integrated into *rulB*-like genes.

The site-specific insertion of ILEs carrying adaptive traits into the *rulB* locus is key to the overall significance of this study as it signifies a potential hotspot for integration of what appear to be atypical integrons that are not primarily associated with acquisition and carriage of antibiotic resistance cassettes (see Cambray *et al*., 2010). Typically, integrons are gene capture systems that comprise a core stable platform of an *intI* gene (a tyrosine recombinase) with its own promoter (P_int_), and an outward facing promoter (P_c_) that can express captured cassettes, and an adjacent upstream *attI* recombination site (Cambray *et al*., 2010) into which cassettes are captured by recombination with the cassette *attC* site. The ILEs described here differ to this typical structure. First, the *intI*-like gene (ORF1; *xerD*) does not appear to contain promoters P_int_ or P_c_, and even if they were present, the gene is oriented in the opposite direction to typical integrons so that Pc would have no effect on expression of the genes in the ‘variable side’ of the ILE. Second, the orientation of *xerD* in ILEs suggests that the *attI* site would be in the region where integration into *rulB* occurs. However, we could not find any such *attI* recombination site adjacent to *xerD* or elsewhere in these ILEs.

ILEs described here are of two types based on the small sequence differences in the left-hand ‘conserved side’ and different ORFs present in the right-hand ‘variable side’. This variation also appears to reflect the bacterial lineages from which they were derived. ILE_FH1_ and ILE_FH5_ share closest homology with each other, and both originated in host bacteria within the *P. fluorescens* subgroup, while ILE_FH4_ had a different variable side and originated in a *P. gessardi* subgroup host. In ILE_FH1–FH5_, the variation in ORFs carried downstream ORFs 1–3 (the three recombinase family ORFs) was akin to the variation in cassettes carried by typical integrons (see Cambray *et al*., 2010). ORFs downstream of the recombinases in ILE_FH1_ and ILE_FH5_ (ORFs 1–3) shared > 96% homology with counterparts on plasmid pGRT1, whereas ORFs in ILE_FH4_ shared homology with those on *P. aeruginosa* genomic islands PAGI-5 and RGP63, and plasmid pUM505. We have not determined the effects on host fitness resulting from insertion of ILEs into *rulB* on pWW0 beyond UV tolerance assessments as a more encompassing assessment of the wider environmental distribution and traits conferred by ILEs is planned. However, based on the evidence in the literature, it is likely that traits conferred by ILEs are of major significance to plant and animal health. In their report on plasmid pGRT1, Molina and colleagues (2011), assessed traits conferred by several of the ORFs located between ORFs 25 and 36 (the region nearly identical to the ILE_FH1_), and showed that some conferred a selective advantage on the host bacterium including the modulation of toluene efflux pump genes located on the chromosome of the host bacterium *P. putida* DOT-1E (see Table 2).

In the genomic island PAGI-5, the region that shared homology with ILE_FH4_ resided within NR-II, which has been shown to make a substantial contribution to the virulence of the host bacterium *P. aeruginosa* PSE9 (Battle *et al*., 2008). In PAGI-5, NR-II spans ORFs 40–62 (approximately 17.5 kb) of which ORFs 49–60 share homology and structural similarities with ILE_FH4_ ORFs 1–11. It is unknown whether the whole 17.5 kb NR-II sequence is required for virulence or whether it is due to a smaller region such as ORFs 49–62 or the ORFs of unknown function (encoding hypothetical proteins) (ORFs 40–48). However, the independent movement and integration of a region with close homology to a key virulence region in animals is extremely significant. This is particularly pertinent when it is considered that similar regions to NR-II were present in six other *P. aeruginosa* PSE strains (PSE11, 15, 17, 30 35 and 39) (Battle *et al*., 2008).

While in the present study, interruption of *rulB* by ILEs in pWW0 was observed in laboratory experiments only, there is evidence that an almost identical *rulB* (*ruvB*) gene on an IncP-9 pWW0-like plasmid, pDTG1, has previously served as an insertion hotspot in the natural environment. Plasmid pDTG1 contains a disrupted *rulB* gene and shares considerable structural and sequence similarity with pWW0, and both are thought to have had a common predecessor (Dennis and Zylstra, 2004). In pDTG1, the *rulB* gene has been disrupted by insertion of a 6 kb region thought to be derived from plasmid pCAR1 and prior to further insertion of genes encoding naphthalene degradation (Dennis and Zylstra, 2004). From sequence analysis of the present newly discovered ILEs and of genomes deposited in databases, we have found no evidence of interruption of the *rulA* gene (or *rulA*-like genes) by insertion. However, *rulB*, or its homologous gene in other bacteria, is frequently seen to be disrupted in other bacterial genomes.

The *rulAB* operon (either intact or interrupted) is often situated close to integrase genes and other fitness/effector/(a)virulence genes in the genomes of pseudomonads. This association extends to *rulAB* relatives such as *rumAB*, *mucAB*, *umuDC* and *samAB* in other genera (see Böltner *et al*., 2002; Dennis and Zylstra, 2004; Li *et al*., 2004; Stavrinides and Guttman, 2004; Sundin *et al*., 2004; Zhao *et al*., 2005; Wozniak *et al*., 2009; Wozniak and Waldor, 2010; Seth-Smith *et al*., 2012). In several of these cases, a *rulB*-like gene (*umuC*, *mucB*, *impB* and *rumB*) is interrupted by a region containing an integrase family gene. Perhaps most noteworthy of these is the SXT-R391 family of integrative and conjugative elements (ICEs) that share 52 core genes as well as five intergenic hotspots for insertion (known as HS1-HS5; see Wozniak *et al*., 2009). Outside of these hotspots are other regions that contain variable DNA. In the cases of the element SXT and the ISCR2-like elements, ICEpdaSpa1, ICEPalBan1, ICEVchInd5, ICEVchBan5, ICEVchBan9/ICEVchMoz10 and ICEVflInd1, the variable regions are inserted into *rumB* (Wozniak *et al*., 2009). None of these elements have relationships with those described here other than that significantly, they reiterate a feature of the *umuC*-encoding subfamily locus in being a hotspot for the insertion of mobile DNAs.

Possible reasons as to why insertion of these ILEs is specific to the *rulB* gene in this case and possibly widespread in nature in *rulB*-like homologues remain unclear. Proteins RulA and RulB are members of the UmuC-like subfamily of lesion-replicating Y-family DNA polymerases (alongside UmuDC, MucAB, ImpAB and RumAB) that are encoded in the chromosomes and plasmids of numerous bacteria. In *Pseudomonas* spp., the role of the *rulAB* operon in the SOS response and the general adaptational traits of the host (Tark *et al*., 2005; Sundin and Weigand, 2007) would suggest that disruption of *rulB* by an insertion event might be detrimental to the host. However, if this interruption did not significantly alter the functionality of RulA or RulB, or the traits acquired by insertion provided a greater fitness benefit than encoded by an intact *rulAB* operon alone, then perhaps selection would be favoured. Interruption of *rulB* at position 6488 on pWW0, as occurred in the present study, did not result in a reduction in UV tolerance (Supporting information Fig. S2). This may suggest that ORF *rulB(2)* encodes a functional protein RulB(2) similar in function to the original RulB (see Fig. 3).

It appears that insertion into *rulB* guarantees some measure of vertical mobility (from chromosome to plasmid within the same host), and this may be extended to horizontal mobility, as more often than not in plasmids (including pWW0), the *rulAB* operon is found close by replication and transfer functions (Gibbon *et al*., 1999).

The presence of conserved features in the left-hand side of the ILEs such as an interrupted *rulB*, a downstream conserved 118–119 bp intergenic region and a conserved *xerD*-like integrase/recombinase followed by two other site-specific recombinase genes may be indicative of a minimum requirement for this integration and resolution. As these ILEs can move from an interrupted chromosomally located *rulB-*like gene into another, it suggests that the *rulB* gene may form part of this minimum region and that homologous recombination may be involved. However, to date, we have been unable to locate regions sequences at the ends or within ILEs that might be evidence of the usual means of insertion such as homologous recombination, transposition and site-specific recombination.

It is important for future studies to determine the mechanisms and driving force behind this movement of ILEs into pWW0 and possibly other loci. We are presently investigating the mechanisms for the movement of ILEs based on evidence that antibiotics (Guerin *et al*., 2009; Guérin *et al*., 2011) and mechanisms of horizontal gene transfer such as conjugation and transformation may trigger the integration of ILEs into *rulB* through induction of the integron integrase (Baharoglu *et al*., 2010; 2012; Cambray *et al*., 2011).

### Concluding remarks

The demonstration here of the active and repeatable integration of related fitness-gene carrying ILEs into *rulB* on pWW0 and the presence of intact *rulAB* (and other UmuC subfamily protein encoding genes) on plasmids and chromosomes suggests that there exists a candidate region in bacteria that can be used to monitor the acquisition and movement of fitness-conferring traits. Additionally, this region might offer a means of capture of novel ecologically and perhaps clinically significant fitness-related elements and allow an understanding of potential virulence, avirulence and fitness-related traits that could impact on plant and animal health. An excellent example of a candidate group with which to test this idea are the pPT23A family plasmids (PFPs) (see Ma *et al*., 2007). This large family contains plasmids harbouring a range of fitness-related genes. In a study of 31 plasmids from this family in pathovars of *P. syringae* (Zhao *et al*., 2005), the full sequence of six PFP plasmids and microarray analysis of 161 genes from the remaining 25 showed that 19 of the 31 contained both *rulA* and *rulB*, and that a further seven contained *rulB* alone (Zhao *et al*., 2005). This study of plasmids from this family and other sources will form the basis of future studies.

## Experimental procedures

### Bacterial strains, plasmids and sampling

Bacterial strains and plasmids are described in Table 1. *Eecherichia coli* strains and *P. putida* PaW340 were maintained on nutrient agar (NA, Oxoid, Basingstoke, UK). Antibiotics used in media were either made up fresh on the day of use or stored at −20°C as 1000× concentration stock solutions.

Environmental isolate FH1 was recovered in 1985 from a laboratory facility in the grounds of the Freshwater Biological Association (Far Sawrey, Cumbria) that received freshwater from Windermere in the English Lake District. Environmental pseudomonads were recovered from sediment/water samples collected in sterile 500 ml bottles in 1995 from Deep Adit, a horizontal drainage shaft that flows into Red Dell Beck from the disused copper mine in Copper Mines Valley (Coniston, Cumbria, UK; National Grid Reference SD290987) (Pickup, 1989). Samples were stored at 4°C for up to 2 days before processing. Pseudomonads were isolated on *Pseudomonas* selective agar (Oxoid, UK) 20°C for up to 5 days, and were purified and maintained on nutrient agar.

### Identification of isolates

All ILE-containing isolates were initially confirmed within the genus *Pseudomonas* by using API 20 NE test strips (Biomerieux, Basingstoke, UK). Deeper phylogenetic placement of selected isolates was carried out based on alignment the *gyrB* gene (Mulet *et al*., 2010) obtained from genome sequencing (see later discussion).

### Colony blotting and DNA hybridization

Colony blots were carried out using the method described by Kobayashi and Bailey (1994).

A 10 kb DNA probe was constructed via digestion of pFBA1001 with *Pst*1 and purification of the restriction fragment after gel electrophoresis using QIAEX II Gel Extraction Kit (Qiagen, Manchester, UK). The probe was labelled with ^32^P-dCTP (GE Healthcare Life Sciences, Buckinghamshire, UK) according to the protocols and using the reagents in the random-primed hexanucleotide labelling kit (Roche, West Sussex, UK).

DNA hybridization was preceded by a prehybridization step carried out in 100 ml (per membrane) prewarmed (68°C) 5 × SSPE [1× SSPE is 0.18 M NaCl, 10 mM NaH2PO4 and 1 mM EDTA (pH 7.7)] containing 5× Denhardt's solution, 0.5% (wt vol^−1^) sodium dodecyl sulphate (SDS), and 0.25% (wt vol^−1^) *N*-lauryl sarcosine and 20 μg ml^−1^ denatured sheared calf thymus DNA for 5 h at 68°C. DNA hybridization was performed in freshly prewarmed hybridization solution (prehybridization solution without the addition of Denhardt's solution) at 68°C for 18–20 h. Unbound radioactive probe DNA was removed by washing membranes twice for 10 min (each time) in 2× SSPE–0.1% (wt vol^−1^) SDS at room temperature (20–25°C) followed by 15 min at 68°C in 1× SSPE–0.1% SDS (w v^−1^) and two washes of 15 min (each) in 0.1× SSPE–0.1% SDS (w v^−1^) at 68°C. The membranes were then wrapped in Clingfilm and exposed to X-ray film (Hyperfilm-MP; GE Healthcare Life Sciences) at −70°C for up to 3 days.

### Conjugation experiments

Filter matings were performed by separately resuspending a loop full of freshly cultured donor and recipient cells in 300 μl 1× phosphate buffered saline (PBS) (pH 7.4) followed by overlaying 10 μl of each suspension on to a 0.22 μm pore size membrane filter (Supor-200, Pall Life Sciences, Portsmouth, UK) on nutrient agar medium and incubation at 28°C (± 0.5°C) for 24 h. Controls (unmixed donors and recipient cells) were treated in the same manner. After incubation, cells and controls were resuspended in 450 μl PBS and transconjugants were selected by spreading onto M9 agar supplemented with the required amino acids and antibiotics to select for transconjugants and against donors and recipients (see Table 1). All transconjugants were confirmed by conferring the required plasmid phenotype in addition to resistance or sensitivity to streptomycin and the requirement for the addition of tryptophan to M9 minimal medium.

Plasmid transfer frequency was determined by growth on M9 medium supplemented with glucose (10 mM) and kanamycin (25 μg ml^−1^), and without the addition of tryptophan (to select against PaW340). Briefly, donor and recipients were cultured in nutrient broth (NB) with antibiotics as required followed by serial dilution in sterile 1× PBS. From these dilutions, spread plating was carried out on non-selective nutrient agar (NA) to determine cell concentrations of donor and recipients. Serially diluted donor and recipient cultures were also mixed (50 μl of each) and spread plated on to selective M9 agar as earlier. Transfer frequency of pWW0 was expressed as transconjugants per recipient cell. Twenty transconjugants from each mating were screened by PCR for the presence of the inserted element using the rulAB-xerDFP and rulAB-xerDRP primer set (see Table 3), and the transfer was expressed as integrations per transconjugant.

### ILE insertion specificity

ILE insertion specificity into *rulB* on pWW0 was investigated by filter matings between strains FH1, FH4 and FH5, and *P. putida* PaW340 host harbouring a plasmid (pWW0Δ*rulAB*::Km^R^) from which 963 bp of *rulAB* (position 6072–7034) had been replaced by a kanamycin resistance gene (Tark *et al*., 2005). Strain PaW340 (pWW0Δ*rulAB*::Km^R^) was constructed by conjugation from original host *P. putida* PaW85 (trp+, Sm^S^) to *P. putida* PaW340 (trp−, Sm^R^).

### ILE insertion frequency

The frequency of ILE integration into *rulB* was assessed by PCR amplification of the rulB-xerD (Table 3) region in 20 confirmed transconjugants after cell lysis at 95°C in sterile 1× PBS. Cell lysis was confirmed in each case by amplification of the xerD region from transconjugants. Frequency of integration was expressed as percentage of rulB-xerD positives to xerD positives.

### UV tolerance assessments

UV tolerance experiments were carried out using a similar method to that of Molina and colleagues (2011). The strains *P. putida* PaW340, *P. putida* PaW340 (pWW0::Km^R^) *P. putida* PaW340 (pWW0::Km^R^::ILE_FH1_ and *P. putida* PaW340 (pWW0::Km^R^::ILE_FH4_) were inoculated into isosensitest broth (supplemented with 25 μg ml^−1^ kanamycin where required for plasmid selection) and cultured at 30°C with shaking overnight. The concentration of cultures was normalized with sterile 1× PBS after absorbance measurements at 280 nm using the Nanodrop ND-1000 and 3 μl of serially diluted suspension (to 10^−5^) were spotted directly onto isosensitest agar plates. Drops were allowed to dry (within 30 min) before direct exposure to UV light. Exposure was carried out using UVP high-performance transilluminator with a 302 nm light source. Prior to incubation at 30°C, plates were inverted and directly exposed to UV at a distance of 1 cm at 15 s intervals up to 1 min. Control plates were not exposed to UV. Three independent assays were carried out with duplicate plates in each.

### Plasmid extraction

Plasmid DNA was extracted from control strains and transconjugants after growth in the required selective media at 30°C with shaking at 150× r.p.m. for 18 h using QIAGEN mini and midi columns (Qiagen, UK).

### PCR amplifications

PCR amplifications were carried out in individual thin-walled 0.2 ml tubes on a Veriti thermal cycler (Life Technologies, Paisley, UK). PCR primers were designed using the Primer 3 software (http://primer3.wi.mit.edu/) (Untergasser *et al*., 2012) (Table 3). Amplified DNA was visualized by agarose gel electrophoresis in gels stained with ethidium bromide and excised from the gel using the Qiagen gel extraction kit II (Qiagen, UK).

### DNA sequencing, annotation and analysis

PCR products were purified using QIAquick PCR purification kit (Qiagen, UK) and sequenced on the top strand directly from the forward primer of the reaction using Qiagen genomic services (Qiagen, Düsseldorf, Germany).

The 10 kb region of pWW0::ILE_FH1_ in pFBA1001 was sequenced commercially (Qiagen, Germany) by Dye Terminator cycle sequencing (using a Model 3730XL automated DNA Analyser; Life Technologies) of pUC19-based shotgun clones to at least six times coverage and accuracy assured to at least 99.995%.

The draft genomes of strains FH1, FH4 and FH5 were sequenced using the Illumina HiSeq platform (Illumina, Cambridge, UK). *De novo* assembly was performed using Velvet with settings selected using VelvetOptimiser (http://www.vicbioinformatics.com/software.velvetoptimiser.shtml). DNA (BLASTn) and protein (BLASTp) alignments, and ORF analysis (ORF Finder) were carried out using NCBI suite of facilities (http://www.ncbi.nlm.nih.gov). Multiple sequence alignments were performed and annotated using CLUSTALW (Thompson *et al*., 2002). Phylogenetic tree construction was carried out using the ‘One Click’ mode within the facilities found at http://www.phylogeny.fr (Dereeper *et al*., 2008; 2010). Graphical representations of DNA were performed manually or using SnapGene V1.4 software (http://www.snapgene.com).

### Nucleotide sequence accession numbers

The DNA sequence of the 10.1 kb region of plasmid pFBA1001 has been deposited at DDBJ/EMBL/GenBank under the accession number KC581795. The Whole Genome Shotgun project data for strains FH1, FH4 and FH5 have been deposited at DDBJ/EMBL/GenBank under the accession numbers AOHM00000000, AOHN00000000 and AOJA00000000 respectively. The versions described in this paper are versions AOHM01000000, AOHN01000000 and AOJA01000000 respectively.

## Supporting information

**Fig. S1.** ClustalW multiple alignment of the predicted protein sequence encoded by ILE_FH1,FH4 & FH5_ ORF1 (XerD recombinase) with closest relatives. The position of conserved C-terminal R-H-R-Y motif of tyrosine recombinases is highlighted in enlarged text.

**Fig. S2.** Survival of *P. putida* PaW340, *P. putida* PaW340 (pWW0::Km^R^), *P. putida* PaW340 (pWW0::Km^R^::ILE_FH1_), *P. putida* PaW340 (pWW0::Km^R^::ILE_FH4_) and *P. putida* PaW340 (pWW0Δ*rulAB*::Km^R^) after exposure to ultraviolet light. Cell concentrations were normalized and serially diluted to 10^−6^ and spotted onto isosensitest agar. Dried plates were exposed directly to a 302 nm UV source at 15 s intervals for 1 min and incubated overnight at 30°C. Results for 30 s of exposure are shown alongside control plates that were not exposed to UV.

**Fig. S3.** Multiple sequence alignment of 123 bp intact and ILE integrated pWW0 *rulB* with closest relatives (as determined through BLASTn alignment) highlighting the 5′-GAT-3′ insertion region. (A) Intact *rulB* with GAT at position 6488, (B) the ILE-*rulB'* junction at the *xerC/D* side of the ILE, (C) the ILE-*rulB'* junction at the variable gene side of the ILE.

**Fig. S4.** Phylogenetic tree of *Pseduomonas* sp. strains FH1, FH4 and FH5 based upon partial sequence of the *gyrB* gene. Dendrograms were generated using the maximum-likelihood method implemented in the PhyML program (v3.0 aLRT) within the suite of facilities at http://www.phylogeny.fr.

**Fig. S5.** Phylogenetic tree of ORF1 (A), ORF2 (B) and ORF3 (C) (integrase recombinase genes) from ILE_FH1_, ILE_FH4_ and ILE_FH5_. Dendrograms were generated using the maximum-likelihood method implemented in the PhyML program (v3.0 aLRT) within the suite of facilities at http://www.phylogeny.fr.
