# Supplementary material for: The rulB gene of plasmid pWW0 is a hotspot for the site-specific insertion of integron-like elements found in the chromosomes of environmental Pseudomonas fluorescens group bacteria
Source: Environ Microbiol. 2014 Jan 7;16(8):2374–88. doi: 10.1111/1462-2920.12345 (PMC4542609; doi:10.1111/1462-2920.12345)
Supplement: Supplementary file 1 — Fig. S1. ClustalW multiple alignment of the predicted protein sequence encoded by ILEFH1,FH4 & FH5 ORF1 (XerD recombinase) with closest relatives. The position of conserved C-terminal R-H-R-Y motif of tyrosine recombinases is highlighted in enlarged text. [file emi0016-2374-sd1.pdf]

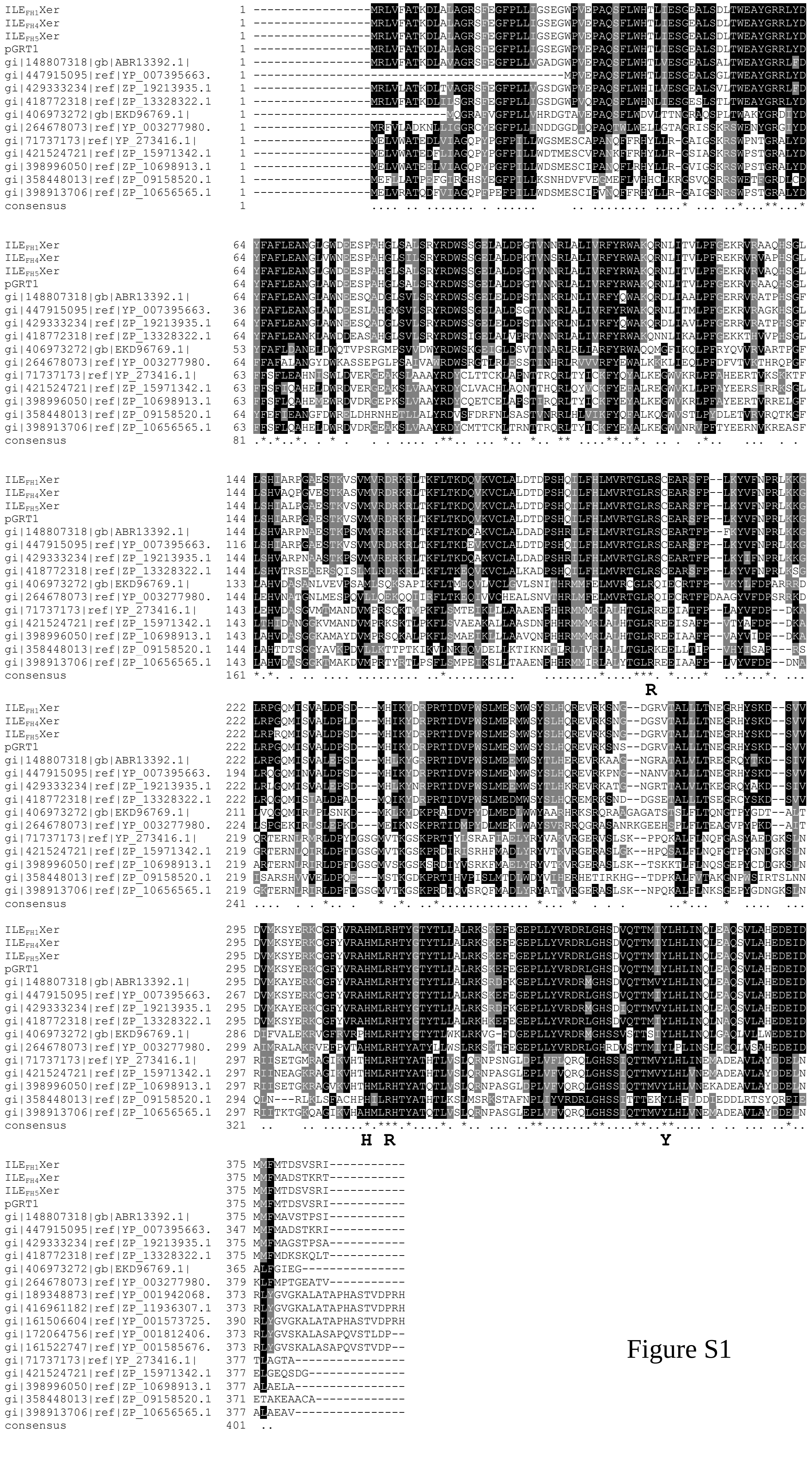

ILE$_{FH1}$Xer
ILE$_{FH4}$Xer
ILE$_{FH5}$Xer
pGRT1
gi|148807318|gb|ABR13392.1|
gi|447915095|ref|YP_007395663.
gi|429333234|ref|ZP_19213935.1
gi|418772318|ref|ZP_13328322.1
gi|406973272|gb|EKD96769.1|
gi|264678073|ref|YP_003277980.
gi|71737173|ref|YP_273416.1|
gi|421524721|ref|ZP_15971342.1
gi|398996050|ref|ZP_10698913.1
gi|358448013|ref|ZP_09158520.1
gi|398913706|ref|ZP_10656565.1
gi|189348873|ref|YP_001942068.
gi|416961182|ref|ZP_11936307.1
gi|161506604|ref|YP_001573725.
gi|172064756|ref|YP_001812406.
gi|161522747|ref|YP_001585676.
consensus
1
64
81
144
161
222
241
295
321
375
401
R
H R
Y

Figure S1
