# Supplementary figures and images for: The rulB gene of plasmid pWW0 is a hotspot for the site-specific insertion of integron-like elements found in the chromosomes of environmental Pseudomonas fluorescens group bacteria
Source: Environ Microbiol. 2014 Jan 7;16(8):2374–88. doi: 10.1111/1462-2920.12345 (PMC4542609; doi:10.1111/1462-2920.12345)

Figure S2

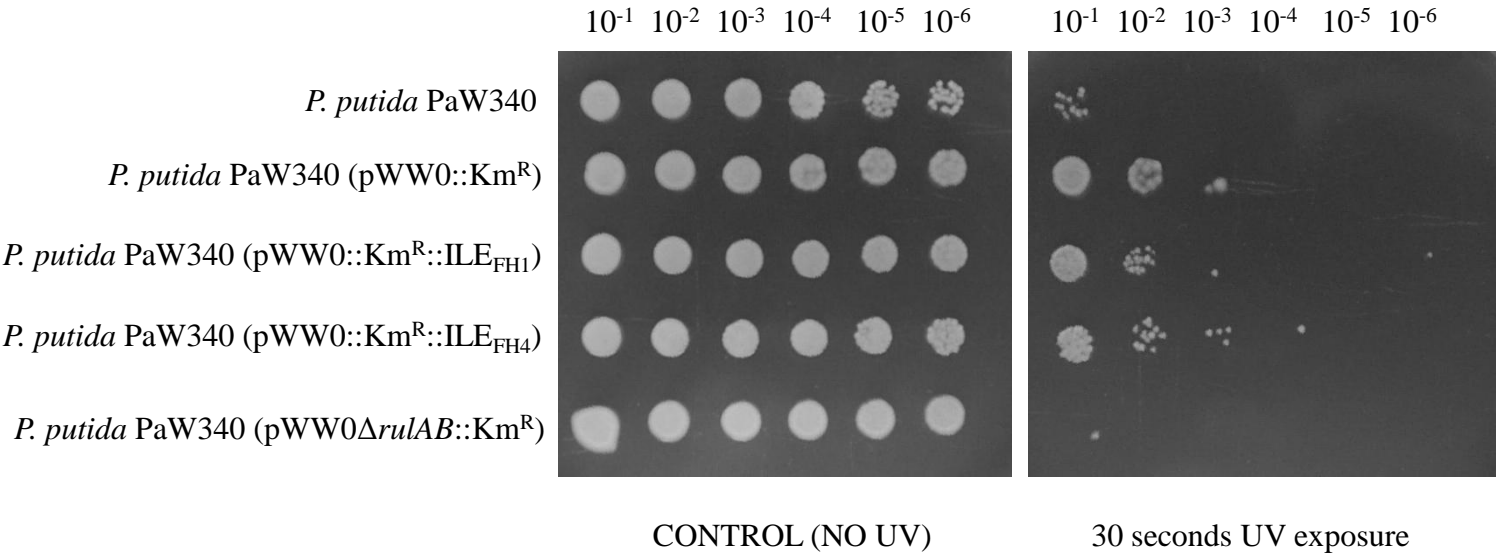

Supplement: Supplementary file 2 — Fig. S2. Survival of P. putida PaW340, P. putida PaW340 (pWW0::KmR), P. putida PaW340 (pWW0::KmR::ILEFH1), P. putida PaW340 (pWW0::KmR::ILEFH4) and P. putida PaW340 (pWW0ΔrulAB::KmR) after exposure to ultraviolet light. Cell concentrations were normalized and serially diluted to 10−6 and spotted onto isosensitest agar. Dried plates were exposed directly to a 302 nm UV source at 15 s intervals for 1 min and incubated overnight at 30°C. Results for 30 s of exposure are shown alongside control plates that were not exposed to UV. [file emi0016-2374-sd2.pdf]

Figure S4

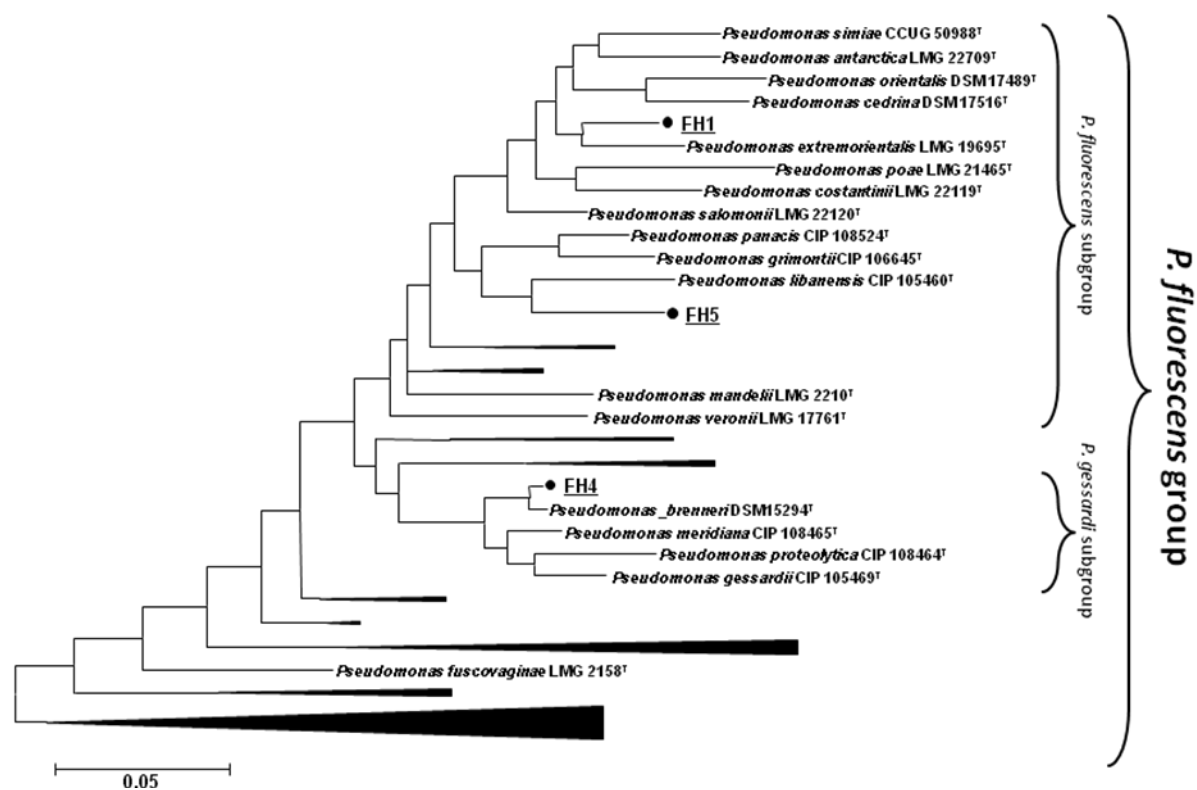

Supplement: Supplementary file 4 — Fig. S4. Phylogenetic tree of Pseduomonas sp. strains FH1, FH4 and FH5 based upon partial sequence of the gyrB gene. Dendrograms were generated using the maximum-likelihood method implemented in the PhyML program (v3.0 aLRT) within the suite of facilities at http://www.phylogeny.fr. [file emi0016-2374-sd4.pdf]

Figure S5

A

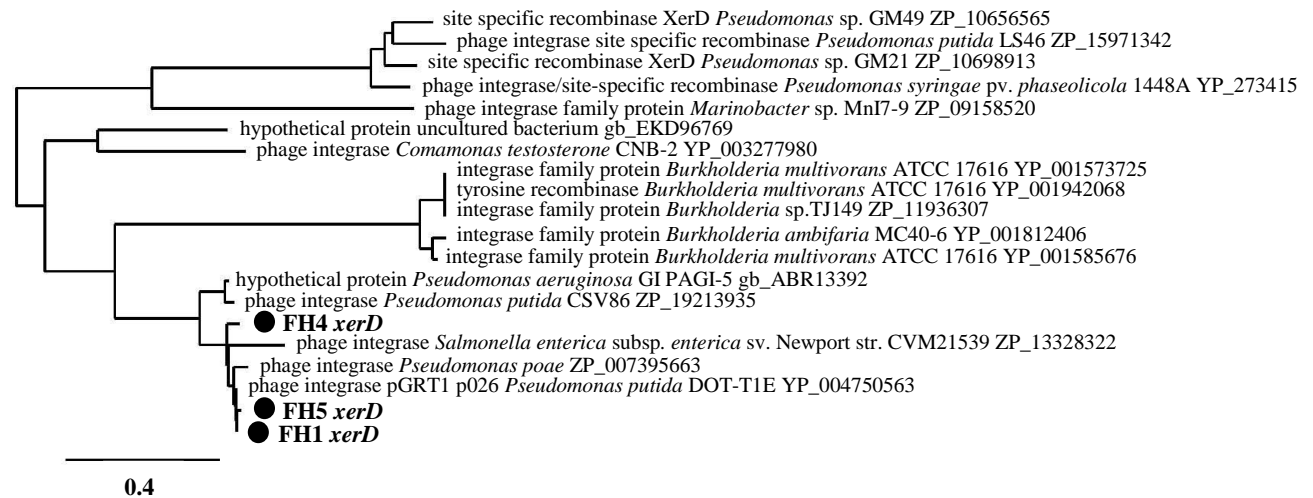

B

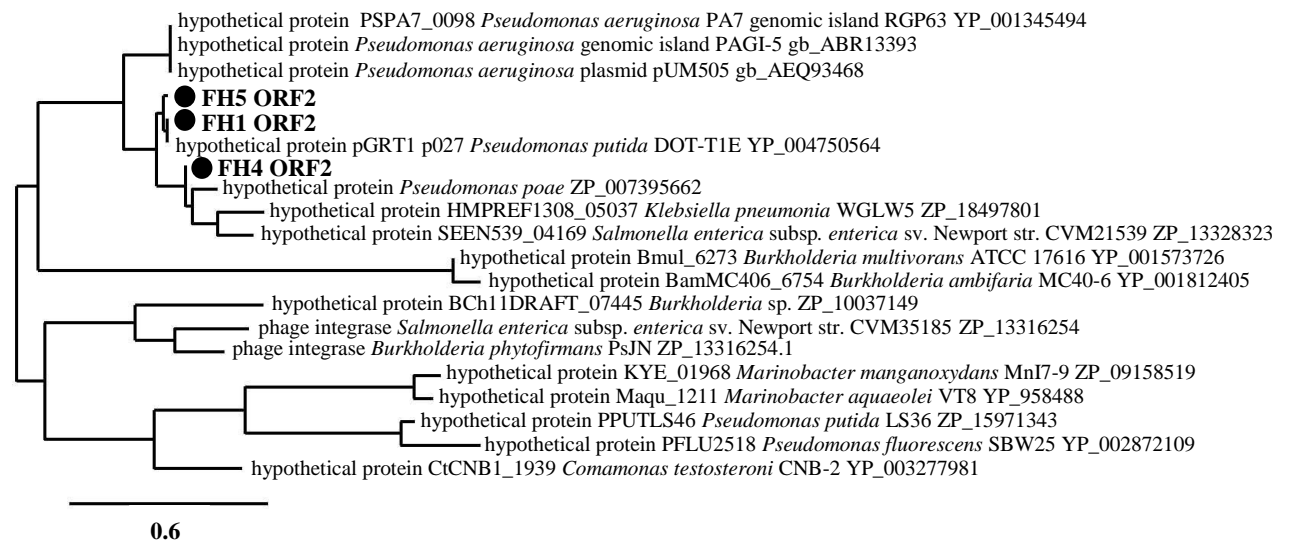

C

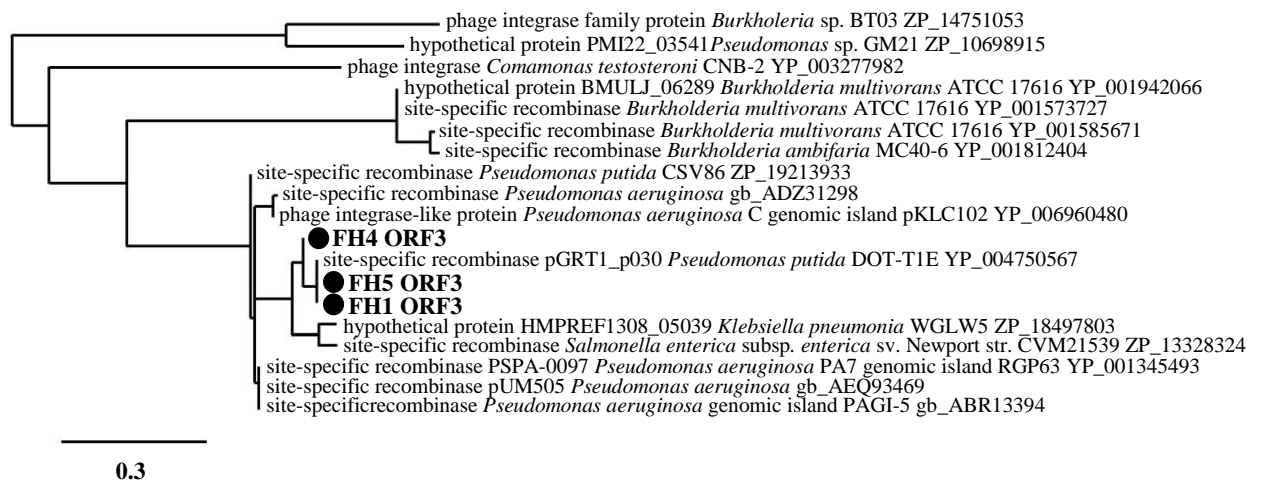

Supplement: Supplementary file 5 — Fig. S5. Phylogenetic tree of ORF1 (A), ORF2 (B) and ORF3 (C) (integrase recombinase genes) from ILEFH1, ILEFH4 and ILEFH5. Dendrograms were generated using the maximum-likelihood method implemented in the PhyML program (v3.0 aLRT) within the suite of facilities at http://www.phylogeny.fr. [file emi0016-2374-sd5.pdf]
