# Supplementary material for: The rulB gene of plasmid pWW0 is a hotspot for the site-specific insertion of integron-like elements found in the chromosomes of environmental Pseudomonas fluorescens group bacteria
Source: Environ Microbiol. 2014 Jan 7;16(8):2374–88. doi: 10.1111/1462-2920.12345 (PMC4542609; doi:10.1111/1462-2920.12345)
Supplement: Supplementary file 3 — Fig. S3. Multiple sequence alignment of 123 bp intact and ILE integrated pWW0 rulB with closest relatives (as determined through BLASTn alignment) highlighting the 5′-GAT-3′ insertion region. (A) Intact rulB with GAT at position 6488, (B) the ILE-rulB' junction at the xerC/D side of the ILE, (C) the ILE-rulB' junction at the variable gene side of the ILE. [file emi0016-2374-sd3.pdf]

Figure S3

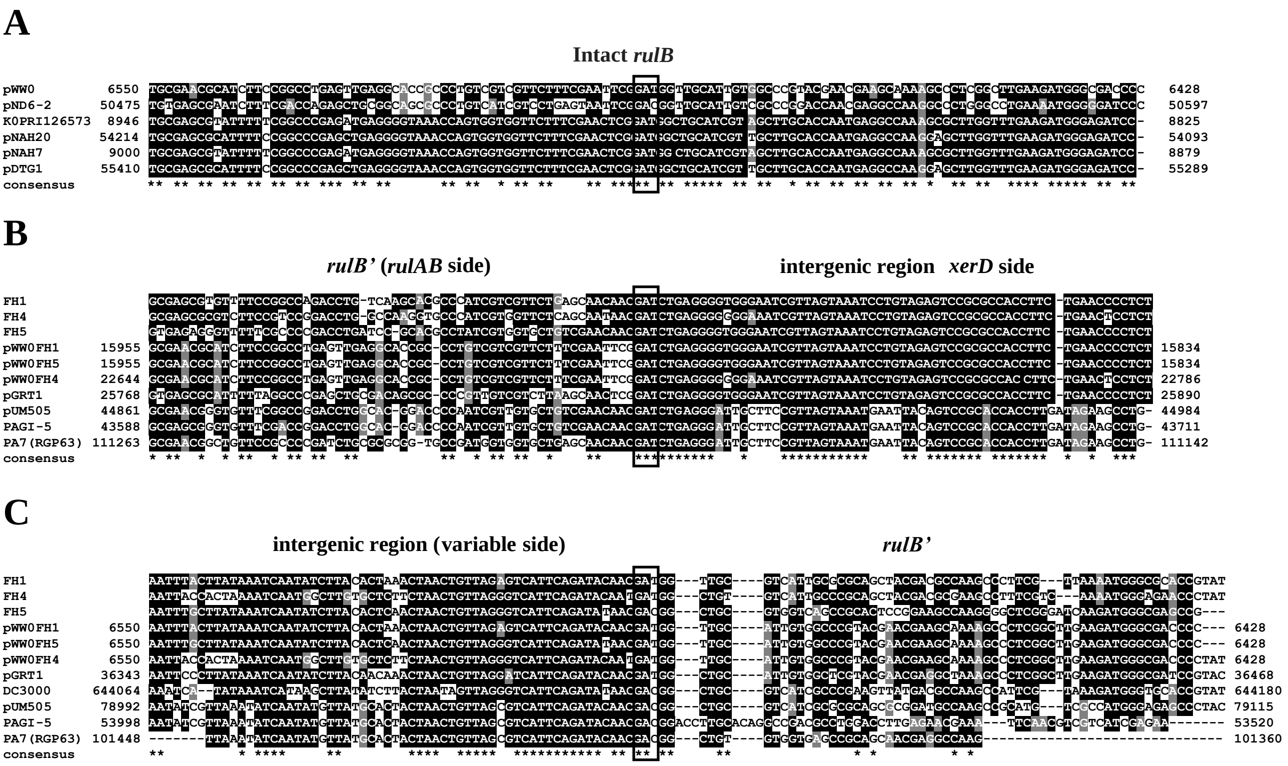
A
Intact rulB
B
rulB' (rulAB side)
intergenic region xerD side
C
intergenic region (variable side)
rulB'
